# Aldose Reductase and Protein Glycation Inhibitory Activity of Dark Chocolate-Assisted Zinc Oxide Nanoparticles

**DOI:** 10.7759/cureus.48953

**Published:** 2023-11-17

**Authors:** Vedha R Nair, Geetha R V, Parameswari R P

**Affiliations:** 1 Microbiology, Saveetha Dental College and Hospitals, Saveetha Institute of Medical and Technical Sciences, Saveetha University, Chennai, IND; 2 Pharmacology, Centre for Transdisciplinary Research, Saveetha Dental College and Hospitals, Saveetha Institute of Medical and Technial Sciences, Saveetha University, Chennai, IND

**Keywords:** zinc oxide, green synthesis, nanoparticles, protein glycation, aldose reductase, diabetes mellitus

## Abstract

Introduction

One of the most common health issues that the global population is dealing with is the associated complications of diabetes, which encompasses cataracts, peripheral neuropathy, vascular damage, impaired wound healing, retinal issues, and arterial wall stiffening. The present study is aimed to evaluate the effect of dark chocolate and its assisted zinc oxide nanoparticles against diabetes-associated complications.

Materials and methods

Zinc oxide nanoparticles were synthesized using commercially dark chocolate (DC-ZnO NP). The synthesized DC-ZnO NPs were evaluated against recombinant aldose reductase (AR) activity and the formation of advanced glycation end products (AGEs). Aminoguanidine and gallic acid were used as reference standards for AGE assay and sorbitol accumulation inhibition, respectively.

Results

The results of the present study showed that green synthesized DC-ZnO NP had a significant dose-dependent inhibitory activity on both AR and AGEs. The inhibitory activity was compared to that of quercetin and aminoguanidine, respectively.

Conclusion

Targeting the endogenous antioxidant systems like AGEs and AR enzymes seems to provide a promising therapeutic approach, thus concluding that ZnO-NP could be a promising agent for treating diabetes-related complications such as diabetic retinopathy, diabetic nephropathy, and diabetic neuropathy that provide grounds for further clinical investigations and trials.

## Introduction

Diabetes mellitus is a broad term encompassing various metabolic conditions, with chronic high blood sugar levels being the most prevalent manifestation. The primary culprits behind diabetes are either insufficient insulin production, impaired insulin function, or a combination of both. In its initial phases, diabetes poses a substantial risk for atherosclerotic vascular disease and coronary heart disease [1]. Diabetes patients have twice the rate of heart disease as the general population. Other associated complications of diabetes are retinopathy, nephropathy, neuropathy, etc. [2]. The global prevalence of diabetes has risen considerably in recent decades, and it is anticipated to continue to rise in the future. Obesity and advancing age are both linked to an increased incidence of diabetes, regardless of race or ethnicity [3]. The stimulation of the sorbitol tract induces non-enzymatic protein glycation, which leads to basement membrane thickening and endothelial cell proliferation. This membrane thickening leads to increased vascular resistance hence reducing tissue perfusion and causing nerve hypoxia [4]. The basis for aldose reductase (AR) inhibition in diabetes is the high AR pathway activity in the peripheral nerve and other tissues that are susceptible to complications related to diabetes, and its activation by hyperglycemia [5].

With a high flavonoid concentration, cocoa beans and derivatives, such as cocoa powder and chocolate, are one of the greatest sources of antioxidants. Several studies have shown that flavonoids present in cocoa have antioxidant and anti-diabetic characteristics that affect glucose metabolism [6]. Many studies have found that flavanol-rich cocoa products can enhance endothelial function, decrease platelet reactivity, increase insulin sensitivity, and lower systolic and diastolic blood pressure whether consumed acutely or on a regular basis [7,8].

Zinc plays a crucial role in various physiological processes, particularly in the regulation of carbohydrate metabolism [9]. It is highly concentrated in the beta cells of the pancreas, where it is involved in the synthesis, storage, crystallization, and release of insulin [10]. A deficiency in zinc is known to exacerbate kidney damage caused by diabetes. Several clinical observations suggest that zinc may have a preventive effect on the development of diabetic heart and kidney problems [11]. Zinc oxide nanoparticles (ZnONPs) represent a novel method for delivering zinc and hold promise for treating various diseases, including diabetes [12]. The development of a zinc-based medication could be beneficial for managing diabetes and its complications, as preclinical trials have shown positive effects of zinc supplementation [13]. Therefore, the aim of this study is to evaluate the AR and protein glycation inhibitory activity of dark chocolate and its assisted zinc oxide nanoparticles.

## Materials and methods

Preparation of dark chocolate extract

About 100 mg of dark chocolate, obtained from a commercial source, was dissolved in 100 ml of distilled water and heated to 50^o^C. Subsequently, the resulting mixture underwent filtration, initially using Whatman filter paper no. 1 and then through a vacuum filter with a pore size of 0.2 µm. The final filtrate was preserved in a cool, dry location for future utilization.

Synthesis of zinc oxide nanoparticles using dark chocolate

About 25 ml of zinc acetate dihydrate (Zn(NO_3_)_2_·2H_2_O) were combined with 4 ml of dark chocolate extract and stirred on a magnetic stirrer at 60°C for two hours. After the reaction was complete, the mixture was cooled to 25°C and then centrifuged at 10,000 rpm for 10 minutes. The liquid portion was discarded, and the remaining solid was washed three times with distilled water. It was then transferred to a clean Petri dish, dried in an oven at 90°C, and subsequently ground into a fine powder using a mortar and pestle. This powder was heated at 500°C for two hours to eliminate any impurities through a process called calcination. The resulting annealed powder was stored in a sealed glass container and was utilized for various biological applications.

Determination of AR inhibition

A total of 531 μL of 0.1 M potassium buffer (pH 7.0), 90 μL of nicotinamide adenine dinucleotide phosphate hydrogen (NADPH) solution (1.6 mM in potassium buffer), 90 μL of recombinant human AR (6.5 U/mg) (SRP6371-100UG, Sigma-Aldrich, St. Louis, United States), 90 μL of ammonium sulfate solution (4 M in potassium buffer), and 90 μL of DL-glyceraldehyde (25 mM in potassium buffer) were mixed with 9 μL of different concentrations of ZnO nanoparticles (5, 10, 20, 40, 80, and 160 µL) in a cuvette, and the activity of AR was assessed spectrophotometrically by measuring the decrease in NADPH absorbance at 340 nm for three minutes using a spectrophotometer (Biotek Synergy H4 multimode reader, Thermo Fisher Scientific Inc., Waltham, United States). Quercetin was used as a positive control. The inhibition of AR (%) was calculated using the following equation: (1 − (△A sample/min) - (△A blank/min)/(△A control/min) − (△A blank/min)) × 100%, where △A sample/min is the decrease in absorbance over three minutes with reaction solution, test sample, and substrate, and △A control/min without the test sample [14].

Advanced glycation end-product assay

Advanced glycation end products (AGEs) [15] are formed by non-enzymatic glycosylation of proteins that enhance vascular permeability in both micro and macro vascular structures by binding to specific macrophage receptors. The ZnO nanoparticles were evaluated for their activity on AGEs formation. AGE reaction mixture was constituted as follows; 1 mg/mL bovine serum albumin (BSA) in 50 mM sodium phosphate buffer (pH 7.4) and 0.02% sodium benzoate into 0.2 M fructose and 0.2 M glucose. The reaction mixture (2.75 mL) was treated with different volumes of ZnO nanoparticles (5, 10, 20, 40, 80, and 160 µg/ml). Amino guanidine was used as a positive control. After incubating at 37°C for three days, the fluorescence intensity of the reaction was determined at excitation and emission wavelengths of 350 nm and 450 nm, respectively, using a Biotek synergy multi-mode reader (Agilent, Santa Clara, United States). The percentage activity was calculated with respect to solvent control.

## Results

Sorbitol accumulation inhibition activity of DC-ZnO NP

**Figure 1:**
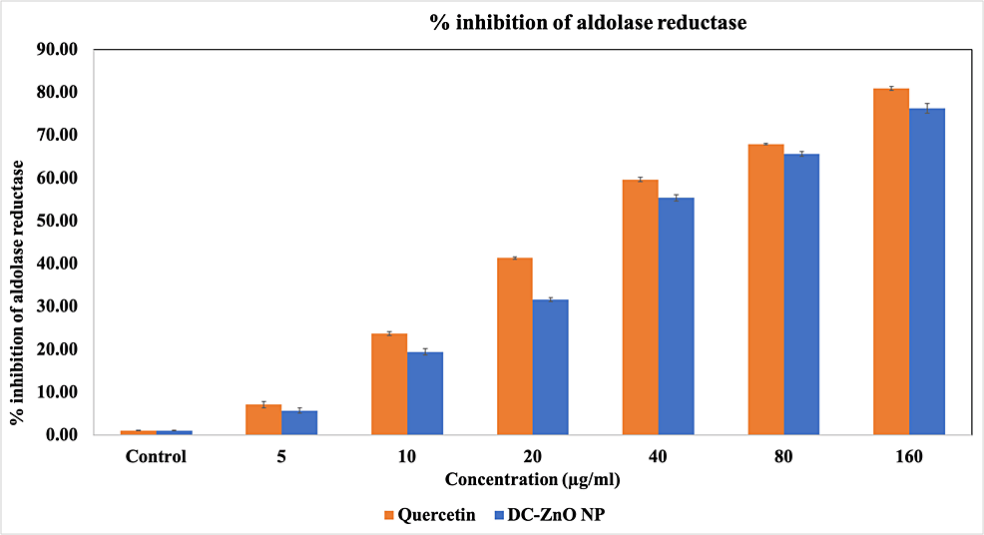
Effect of dark chocolate-mediated zinc oxide nanoparticles (DC-ZnO NP) on Aldolase reductase enzyme activity

In the pathogenesis of diabetic complications, it is thought that an important role is played by increased oxidative stress, as supported by the increased levels of oxidized DNA, lipids, and proteins [16]. Increased AR activity has been linked to the development of intracellular sorbitol accumulation, which was linked to a number of secondary complications associated with diabetes. Inhibiting aldolase reductase may be a useful tactic for delaying or preventing some diabetic problems [17]. Figure *1* shows that there was a dose-dependent increase in the AR inhibition by dark chocolate-mediated ZnONP which was comparable to the standard quercetin.

Inhibitory activity of DC-ZnO NPs on advanced glycation end products

**Figure 2:**
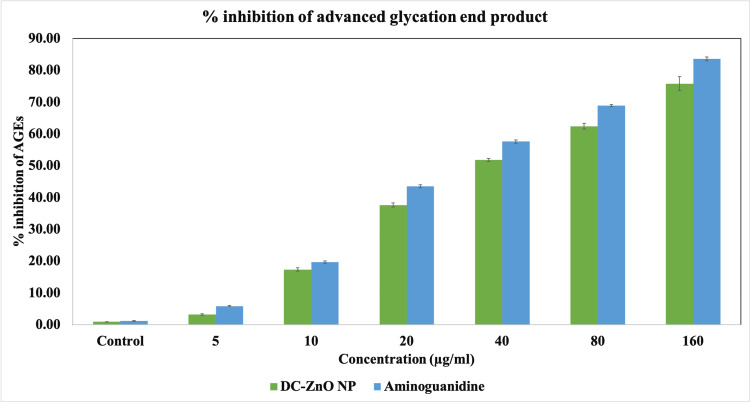
Inhibitory effect of dark chocolate mediated zinc oxide nanoparticles (DC-ZnO NP) on advanced glycation end product (AGE)

The formation of AGEs is a process that can result in the production of reactive oxygen species (ROS). Additionally, when AGEs interact with their receptors, known as receptor for AGEs, it can also lead to the generation of ROS. These AGEs tend to accumulate in various tissues affected by diabetic complications, such as the kidneys, retina, and atherosclerotic plaques. The glycation of proteins disrupts their normal functions by affecting molecular interactions, altering enzymatic activity, reducing their degradation capacity, and interfering with receptor recognition [15]. Figure *2* in the study indicates that the presence of dark chocolate-mediated zinc oxide nanoparticles led to a dose-dependent reduction in the formation of AGEs. This suggests that these nanoparticles have the potential to effectively mitigate complications associated with diabetes.

## Discussion

The current study's findings suggest that inhibiting the AR enzyme and AGEs can decrease the risk of diabetes-related complications. In vivo experiments provide strong evidence that high blood sugar levels contribute to oxidative stress by generating ROS, leading to acute dysfunction of the blood vessel endothelium in diabetic patients [16]. The research proposes that excessive production of superoxide due to mitochondrial electron transport triggered by hyperglycemia plays a central role in initiating various pathways responsible for the development of complications associated with diabetes. These pathways include non-enzymatic glycation, hexosamine pathway activation, protein kinase activity, and mitochondrial respiratory chain [17-19].

Excessive generation of ROS disrupts cellular signaling pathways and overall cellular balance, leading to instability in pancreatic beta cells and increased insulin resistance. This resistance to insulin is a crucial factor in the development of elevated blood sugar levels, contributing to complications in both small and large blood vessels associated with diabetes [20, 21]. In a study conducted by Asri and colleagues, administering zinc oxide nanoparticles (ZnO-NP) at a dose of 3 mg/kg/day for eight weeks resulted in significant reductions. Specifically, it halved brain natriuretic peptide levels, decreased the atherogenic index by 80%, and lowered serum cholesterol levels in diabetic rats [22]. Another study by Nazarizadeh and his team concluded that ZnO-NP played a role in restoring catalase activity in both serum and erythrocytes of diabetic rats [23]. Furthermore, results from oral glucose tolerance tests suggested that ZnO-NP could potentially improve glucose tolerance in experimental diabetes [24]. The extent of this effect varied in a dose-dependent manner, with concentrations of 10 mg/kg/day reducing it to approximately 40% to 70% compared to the diabetic control group [25].

Abd El-Khalik et al. conducted a study proposing the potential of ZnO-NPs to mitigate diabetic nephropathy progression by influencing the interplay between autophagy and the Nrf2/TXNIP/NLRP3 inflammasome signaling pathways [26]. In an earlier investigation, Jan et al. explored the therapeutic effects of ZnO-NPs derived from Aquilegia pubiflora. Administered at a dosage of 200mg/ml, these nanoparticles exhibited significant inhibition of alpha-glucosidase and alpha-amylase, underscoring their anti-diabetic properties [27]. Another study examined the use of green-synthesized ZnO-NPs, combined with natural polymers and other therapeutic agents, to address delayed wound healing in diabetic individuals. This approach demonstrated efficient antibacterial activity against diabetic wound infections, accelerating the wound-healing process [28]. Additionally, ZnO-NPs synthesized from Morus indica significantly inhibited methylglyoxal-mediated glycation of BSA, showing a dose-dependent inhibition of AGEs formation [29]. Recent findings have also shown that ZnO-NPs markedly prevent AGE formation in diabetic mice by acting on hypoglycemic and antihyperlipidemic pathways [30,31]. Furthermore, ZnO-NPs not only prevent AGE formation but also inhibit protein structure changes, thereby hindering AGE formation. Moreover, the capacity of ZnO-NPs to mitigate oxidative stress, particularly by targeting advanced glycation end products, not only holds potential benefits for managing diabetes-related complications but also shows promise in addressing neurodegenerative disorders and other degenerative conditions [32].

The glycation reaction sets off a harmful cycle as it interacts with free radicals and carbonyl species, ultimately resulting in the generation of AGEs. This process significantly contributes to the onset and progression of various diseases. In light of the detrimental impact of AGEs, numerous anti-glycating agents have been identified [32]. Nevertheless, there is an urgent need to explore more effective agents to address this serious clinical issue. According to the given findings, zinc oxide, renowned for its antioxidant properties, has demonstrated potential in inhibiting AGE formation [26-31]. However, it is crucial to adopt a systematic and mechanistic approach before the significant role of ZnO-NPs can be effectively harnessed in the medical field.

Limitations

The initial preclinical evaluation provides preliminary data indicating the impact of synthesized nanoparticles on complications related to diabetes. Nevertheless, additional research is essential, involving both in vitro studies using cell lines and in vivo investigations focused on assessing toxicity and efficacy. Further studies are required to establish and validate the potential of the synthesized nanoparticles for the treatment of complications associated with diabetes.

## Conclusions

The increasing global prevalence of diabetes mellitus and its associated complications necessitates the search for effective treatments. Nanotechnology and nanomedicine advancements have opened up possibilities for biomedical applications and disease management, including the use of materials like ZnO-NP. In this study, we found that dark chocolate ZnO-NP, synthesized through a green process, exhibited a dose-dependent inhibitory effect on both AR and the formation of protein glycation end products. By targeting the body's natural antioxidant systems, such as AGEs and the AR enzyme, this approach appears to offer a promising therapeutic avenue. In conclusion, ZnO-NP shows great promise as a treatment agent for diabetes and its associated complications, warranting further investigation through clinical trials.
